# Better Life’s Essential 8 contributes to slowing the biological aging process: a cross-sectional study based on NHANES 2007–2010 data

**DOI:** 10.3389/fpubh.2024.1295477

**Published:** 2024-03-13

**Authors:** Dongzhe Wu, Chaoyi Qu, Peng Huang, Xue Geng, Jianhong Zhang, Yulin Shen, Zhijian Rao, Jiexiu Zhao

**Affiliations:** ^1^Exercise Biological Center, China Institute of Sport Science, Beijing, China; ^2^Physical Education College, Hebei Normal University, Shijiazhuang, China; ^3^National Institute of Sports Medicine, Beijing, China; ^4^College of Physical Education, Shanghai Normal University, Shanghai, China

**Keywords:** Life’s Essential 8, National Health and Nutrition Examination Survey, Phenotypic Age Acceleration, biological aging, healthy lifestyle

## Abstract

**Objective:**

To investigate the relationship between Life’s Essential 8 (LE8) and Phenotypic Age Acceleration (PhenoAgeAccel) in United States adults and to explore the impact of LE8 on phenotypic biological aging, thereby providing references for public health policies and health education.

**Methods:**

Utilizing data from the National Health and Nutrition Examination Survey (NHANES) conducted between 2007 and 2010, this cross-sectional study analyzed 7,339 adults aged 20 and above. Comprehensive assessments of LE8, PhenoAgeAccel, and research covariates were achieved through the integration of Demographics Data, Dietary Data, Laboratory Data, and Questionnaire Data derived from NHANES. Weighted generalized linear regression models and restricted cubic spline plots were employed to analyze the linear and non-linear associations between LE8 and PhenoAgeAccel, along with gender subgroup analysis and interaction effect testing.

**Results:**

(1) Dividing the 2007–2010 NHANES cohort into quartiles based on LE8 unveiled significant disparities in age, gender, race, body mass index, education level, marital status, poverty-income ratio, smoking and drinking statuses, diabetes, hypertension, hyperlipidemia, phenotypic age, PhenoAgeAccel, and various biological markers (*p* < 0.05). Mean cell volume demonstrated no intergroup differences (*p* > 0.05). (2) The generalized linear regression weighted models revealed a more pronounced negative correlation between higher quartiles of LE8 (Q2, Q3, and Q4) and PhenoAgeAccel compared to the lowest LE8 quartile in both crude and fully adjusted models (*p* < 0.05). This trend was statistically significant (*p* < 0.001) in the full adjustment model. Gender subgroup analysis within the fully adjusted models exhibited a significant negative relationship between LE8 and PhenoAgeAccel in both male and female participants, with trend tests demonstrating significant results (*p* < 0.001 for males and *p* = 0.001 for females). (3) Restricted cubic spline (RCS) plots elucidated no significant non-linear trends between LE8 and PhenoAgeAccel overall and in gender subgroups (*p* for non-linear > 0.05). (4) Interaction effect tests denoted no interaction effects between the studied stratified variables such as age, gender, race, education level, and marital status on the relationship between LE8 and PhenoAgeAccel (*p* for interaction > 0.05). However, body mass index and diabetes manifested interaction effects (*p* for interaction < 0.05), suggesting that the influence of LE8 on PhenoAgeAccel might vary depending on an individual’s BMI and diabetes status.

**Conclusion:**

This study, based on NHANES data from 2007–2010, has revealed a significant negative correlation between LE8 and PhenoAgeAccel, emphasizing the importance of maintaining a healthy lifestyle in slowing down the biological aging process. Despite the limitations posed by the study’s design and geographical constraints, these findings provide a scientific basis for the development of public health policies focused on healthy lifestyle practices. Future research should further investigate the causal mechanisms underlying the relationship between LE8 and PhenoAgeAccel and consider cross-cultural comparisons to enhance our understanding of healthy aging.

## Introduction

With the extension of human lifespan, aging emerges as a significant public health challenge, engendering a slew of health issues including increased susceptibility to chronic diseases and diminished quality of life. This scenario necessitates a pivot toward understanding and alleviating the aging process, with biological aging—a gradual decline in the functionality of bodily tissues and organs increasing susceptibility to diseases—standing as a pivotal area of study (1, 2). The formulation of preventative strategies and interventions against aging is urgent to foster the progression of healthy aging. Noteworthy is the considerable variation in the pace of aging across individuals, a disparity that mirrors directly in their vulnerability to mortality and morbidity. Hence, distinguishing the individual aging velocities, especially early in the lifecycle, is vital, aiding in the early identification of high-risk individuals or potential high-risk groups, thereby offering strategic guidance and support for more precise secondary and tertiary preventions (3).

In recent years, researchers have ventured deeper into the interrelations between biological aging and a myriad of health indicators encompassing biomarkers, diet, physical activity, and disease risks (1). Epigenetics presents a promising avenue in this context, offering a lucrative method to gauge and comprehend biological age and the velocity of aging, known as epigenetic age (4), an approach amalgamating various biomarkers to efficaciously predict the risks associated with mortality, cancer, cardiovascular diseases, and other health issues (5–8).

To foster a robust understanding of the aging process and its influencers, this study introduces two pivotal metrics: Life’s Essential 8 (LE8) and Phenotypic Age Acceleration (PhenoAgeAccel). LE8, delineated by the American Heart Association, encapsulates a cohort of crucial metrics devoted to enhancing and sustaining cardiovascular health, encompassing two primary domains of healthy behaviors and health factors, addressing individual aspects like diet, physical activity, and nicotine exposure, aiming to mitigate the risks of heart diseases, strokes, and other principal health issues (9, 10). PhenoAgeAccel, an emergent aging measurement technique grounded in epigenetics, offers a more authentic reflection of an individual’s biological age, superseding mere chronological age computation (1, 3, 11–16). This method has manifested substantial predictive value, having been employed to prognosticate a range of health outcomes and chronic disease risks (17–20).

Specifically, detrimental dietary habits and low levels of physical activity have been pinpointed as critical accelerators of aging, while moderate alcohol consumption and avoidance of smoking have been associated with a slower aging trajectory (21–23). Recent studies have further illuminated the associations between sleep, prolonged sitting, physical activity, and PhenoAgeAccel, accentuating the bearing of lifestyle on the aging process (19).

Given the above, this research seeks to delve deep into the relationship between LE8 and PhenoAgeAccel, endeavoring to elucidate how LE8 influences the process of phenotypic biological aging. The preliminary hypothesis of this study posits a significant inverse association between LE8 and PhenoAgeAccel, suggesting that healthier lifestyles may decelerate the biological aging process to an extent. Through a rigorous exploration of the interrelation between these variables, the study not only aspires to unravel the biological mechanisms underlying aging but also aims to furnish more targeted guidance for public health policies and health education.

## Methods

### Study subjects and data sources

This study utilized data from the National Health and Nutrition Examination Survey (NHANES) conducted from 2007 to 2010. NHANES is a nationally representative survey executed by the United States Centers for Disease Control and Prevention (CDC) aimed at evaluating the health and nutritional status of adults and children in the United States. The participants were primarily derived from the NHANES database, adhering to the guidance principles of the Strengthening the Reporting of Observational Studies in Epidemiology (STROBE). This biennial survey, executed by the National Center for Health Statistics and the CDC, employs a multistage probability sampling design to examine about 10,000 non-institutionalized individuals from across the United States. The data collection encompasses household interviews and physical examinations. During the interviews, participants responded to questions regarding demographic, socioeconomic, dietary, and health-related variables, while the physical examinations measured medical, dental, and physiological biochemical indicators.

**Figure 1:**
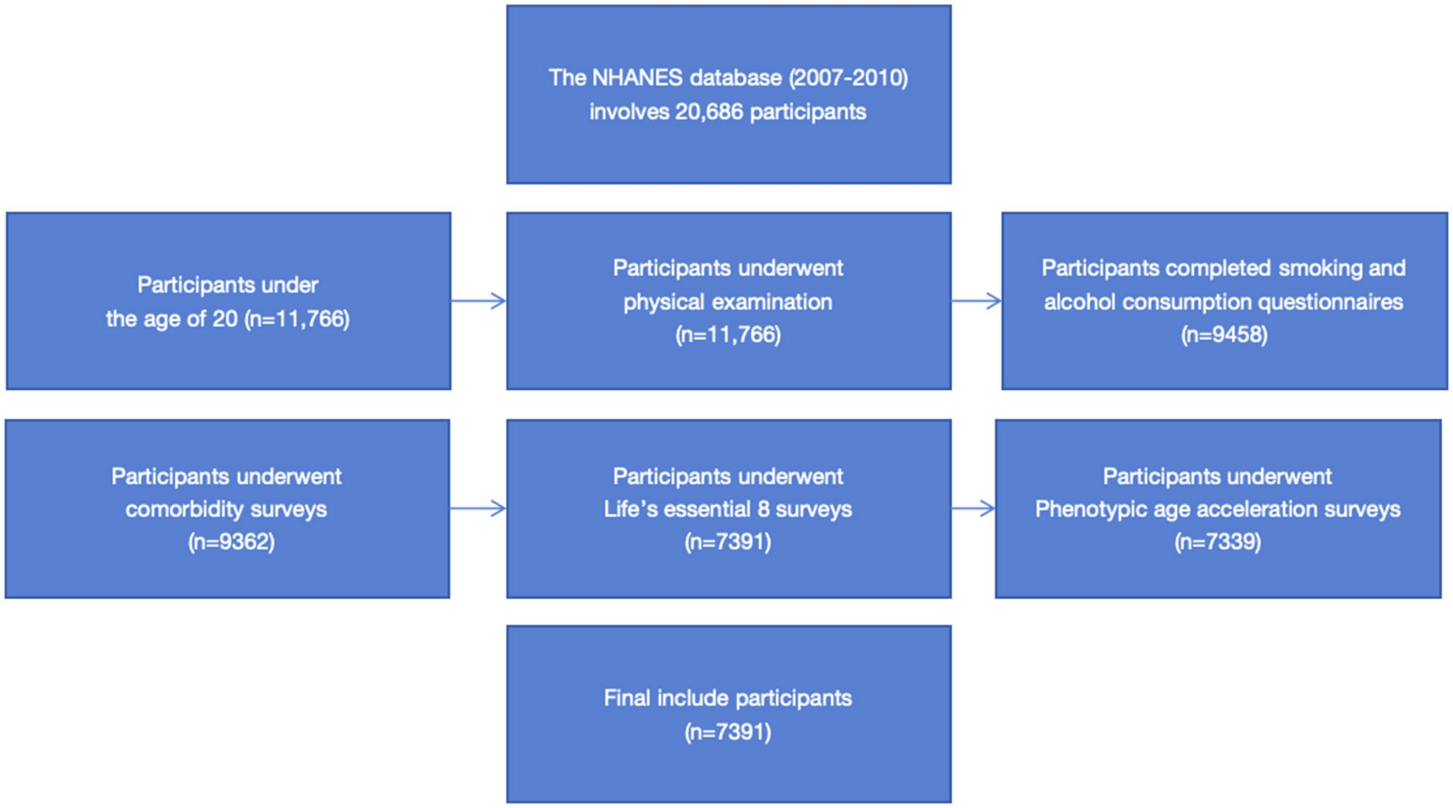
Flow chat.

Between 2007 and 2010, NHANES sampled a total of 20,686 individuals for the survey, of which 11,766 were adults (≥20 years old). We amassed 10,624 valid individual interview questionnaires, excluding 1,142 invalid ones. Out of these, 10,424 participants underwent physical examinations, excluding 200 non-participants; 9,458 completed the smoking and alcohol consumption questionnaires, excluding 966 non-participants; 9,362 were surveyed for comorbidities such as diabetes, hypertension, and hyperlipidemia, excluding 96 non-participants; 7,391 underwent LE8 indicator testing such as physical activity questionnaires, sleep duration questionnaires, and dietary questionnaires, excluding 1,971 non-participants; 7,339 underwent PhenoAgeAccel indicator testing involving laboratory examinations such as blood biochemical data examination, excluding 52 non-participants. Ultimately, we included and retained valid data from 7,339 participants in this research (the detailed screening process is depicted in Figure 1). The survey was conducted in accordance with the Declaration of Helsinki.

### Definitions of Life’s Essential 8

The “Life’s Essential 8” standard set forth by the American Heart Association leverages a series of self-reported questionnaires to assess and score individual health status, encompassing eight dimensions: diet, physical activity, nicotine exposure, sleep health, BMI, blood lipids, blood sugar, and blood pressure (9, 10, 24, 25). Each dimension is categorized into different levels according to specific criteria, with corresponding scores assigned to form a comprehensive score quantifying individual health status.

#### Diet

Dietary information is acquired through a self-administered food frequency questionnaire. Only participants who provided dietary information for 2 days were included in the data analysis, excluding those who provided data for just 1 day. The AHA devised a new methodology to assess dietary quality based on adherence to the “Healthy Eating Index 2015,” segregating populations into different percentile values (1st–24th, 25th–49th, 50th–74th, 75th–94th, and ≥ 95th), corresponding to scores of 0, 25, 50, 80, and 100, respectively (9).

#### Physical activity

Physical activity assessment hinges on the number of minutes of moderate or vigorous physical activity reported weekly by participants through a questionnaire. The duration of physical activity is divided into different levels (0, 1–29, 30–59, 60–89, 90–119, 120–149, and ≥ 150 min/week), with corresponding scores of 0, 20, 40, 60, 80, 90, and 100, respectively (9).

#### Nicotine exposure

The questionnaire gathered self-reported nicotine exposure information from participants. Beyond combustible tobacco use, the AHA also included the use of other nicotine delivery systems such as e-cigarettes and exposure to second-hand smoke in the “Life’s Essential 8” definition. Nicotine exposure is delineated into five categories: current smokers, former smokers (quit <1 year) or current users of inhalable nicotine delivery systems, former smokers (quit 1 to <5 years), former smokers (quit ≥5 years), and never smokers, corresponding to scores of 0, 25, 50, 80, and 100, respectively. An additional 20 points are deducted for adults exposed to indoor smokers (9).

#### Sleep health

Sleep health, a new metric in the “Life’s Essential 8” objectives, is gauged based on the average number of sleep hours reported nightly by participants through a questionnaire. The nightly sleep hours are categorized into different levels (<4, 4 to <5, 5 to <6 or ≥ 10, 6 to <7, 9 to <10, and 7 to <9 h), with respective scores of 0, 20, 40, 70, 90, and 100 (9).

#### Body mass index

Body mass index is calculated using objectively measured weight and height (calculated as the individual’s body weight in kilograms divided by the square of their height in meters.). BMI is segmented into various levels (≥40.0, 35.0–39.9, 30.0–34.9, 25.0–29.9, and < 25.0 kg/m^2^), with corresponding scores of 0, 15, 30, 70, and 100, respectively (9).

#### Blood lipids

Blood samples are utilized to measure total cholesterol and High-Density Lipoprotein (HDL) cholesterol. Non-HDL cholesterol is derived by subtracting HDL cholesterol from the total cholesterol. Non-HDL cholesterol is categorized into different levels (≥220, 190–219, 160–189, 130–159, and < 130 mg/dL), with respective scores of 0, 20, 40, 60, and 100 (9).

#### Blood sugar

Fasting blood samples are employed to measure Fasting Blood Glucose (FBG), along with both fasting and non-fasting blood samples used for HbA1c measurement. Blood sugar levels are segmented into different levels including diabetes (HbA1c ≥10.0%), diabetes (HbA1c 9.0–9.9%), diabetes (HbA1c 8.0–8.9%), diabetes (HbA1c 7.0–7.9%), diabetes (HbA1c <7.0%), non-diabetic but FBG 100–125 mg/dL or HbA1c 5.7–6.4%, and non-diabetic with FBG <100 mg/dL or HbA1c <5.7%. The corresponding scores are 0, 10, 20, 30, 40, 60, and 100, respectively (9).

#### Blood pressure

Blood pressure is measured using a cuff of appropriate size. Blood pressure levels are divided into different categories, including systolic ≥160 mmHg or diastolic ≥100 mmHg, 140–159 or 90–99 mmHg, 130–139 or 80–89 mmHg, 120–129/<80 mmHg, and < 120/80 mmHg, with respective scores of 0, 25, 50, 75, and 100. If undergoing antihypertensive treatment, a deduction of 20 points is applied (9).

### Definitions of Phenotypic Age Acceleration

This study, based on prior representative phenotypic age research, computed individuals’ phenotypic age (1). The phenotypic age is derived through a comprehensive calculation that takes into account the actual age and nine biological markers, including albumin, creatinine, glucose, and C-reactive protein (CRP) represented in logarithmic form, lymphocyte percentage, mean cell volume, red cell distribution width, alkaline phosphatase, and white blood cell count, based on the parameterization of two Gompertz proportional hazard models (where one model utilized all 10 selected variables, and the other solely employed the actual age). These biological markers were determined through a 10-fold cross-validation of mortality using the Cox proportional hazards elastic net model.

In this study, Phenotypic Age Acceleration (PhenoAgeAccel) is ascertained through the calculation of residuals from regressing the phenotypic age against the actual age using a linear model (3). For instance, assuming two individuals are both 50 years old, but one physiologically appears younger, showcasing more youthful health and vitality, while the other seems older due to health issues or adverse lifestyle habits. PhenoAgeAccel, serving as a lower-bound metric where a smaller variable value denotes a slower biological aging process, essentially functions as a marker to gauge an individual’s physiological state relative to their chronological age, aiding in understanding the variations in physiological aging speed relative to the actual age. The specific formula is as follows:


$$\mathrm{Phenotypic}\;\mathrm{Age}=141.50+\frac{\ln\left[-0.00553\times \ln\left(1-xb\right)\right]}{0.09165\text{.}}$$

xb== − 19.907 − 0.0336 × albumin + 0.0095 × creatinine + 0.0195 × glucose + 0.0954 × ln(CRP) − 0.0120 × lymphocyte percent + 0.0268 × mean cell volume + 0.3356 × red cell distribution width + 0.00188 × alkaline phosphatase + 0.0554 × white blood cell count + 0.0804 × chronological age

### Covariate

The covariates included in this study encompass age; gender (male, female); race (Mexican American, non-Hispanic black, non-Hispanic white, and other races); education level (below high school, high school, and above high school); poverty income ratio, calculated by dividing the family (or individual) income by the poverty guidelines set for the survey year (low income with a PIR ≤1.3, medium income with a 1.3 < PIR <3.5, and high income with a PIR ≥3.5); marital status (married/living with a partner, never married, widowed/divorced/separated); body mass index (categorized into <25, 25–29.9, and ≥ 30 kg/m^2^); smoking status categorized as never (smoked less than 100 cigarettes in their lifetime), former (smoked more than 100 cigarettes in their lifetime but not at all now), and current (smoked more than 100 cigarettes in their lifetime and smokes some days or every day); alcohol consumption divided into five categories: never (less than 12 times in their lifetime), former (at least once in the last 12 years but not in the last year, or not in the last year but at least 12 times in their lifetime), light (up to one drink per day for females and up to two drinks per day for males), moderate (up to two drinks per day for females and up to three drinks per day for males), and heavy (up to three drinks per day for females and up to four drinks per day for males); criteria for hypertension diagnosis: (1) being informed by a doctor or healthcare professional of having hypertension, (2) having used antihypertensive drugs, and (3) having a systolic blood pressure of ≥140 mmHg and a diastolic blood pressure of ≥90 mmHg in three measurements; criteria for hyperlipidemia diagnosis: (1) having triglycerides (TG) levels of ≥150 mg/dL, (2) having serum total cholesterol (TC) levels of ≥200 mg/dL, having low-density lipoprotein (LDL) levels of ≥130 mg/dL, having high-density lipoprotein (HDL) levels of <40 mg/dL for males and < 50 mg/dL for females, and (3) using lipid-lowering drugs; criteria for diabetes diagnosis: (1) being informed by a doctor or healthcare professional of having diabetes, (2) having glycated hemoglobin (HbA1c) levels of ≥6.5 mmol/L, (3) having fasting blood glucose (GHLU) levels of ≥7.0 mmol/L, and (4) having used anti-diabetic drugs.

### Statistical methods

This study adhered to the NHANES complex sampling survey procedures, utilizing the complex sampling weights provided in the NHANES analytical guidelines for computation. The analysis of the indicators was conducted using weighted data to yield nationally representative data estimates. Continuous variables involved in this study were presented as mean (standard error), while categorical variables were denoted by actual numbers (weighted percentages). For the intergroup variability test, one-way ANOVA was applied to continuous variables, and the chi-squared test was employed for categorical variables. A weighted generalized linear regression model was adopted to analyze the linear relationship between LE8 and PhenoAgeAccel, further branching into gender sub-group analyses. Moreover, based on the outcomes of the linear regression, non-linear trends between the variables were examined through unrestricted cubic splines. Ultimately, control variables were incorporated into the interaction effect testing model to further verify whether there were interaction effects between the control variables and the relationship between LE8 and PhenoAgeAccel. A threshold of a two-sided *p* < 0.05 was considered statistically significant. All analyses were executed using R Studio (version 4.2.1, United States).

## Results

### Baseline characteristics of the study population

Table 1 displays the characteristics of the study population in the 2007–2010 NHANES, categorized into quartiles based on the LE8 score. Significant differences (*p* < 0.05) were observed across the quartiles for variables such as age, gender, race, body mass index, education level, marital status, poverty income ratio, smoking status, alcohol consumption, diabetes, hypertension, hyperlipidemia, phenotypic age, phenotypic age acceleration, albumin, creatinine, glucose, C-reactive protein, lymphocyte percentage, red cell distribution width, alkaline phosphatase, and white blood cell count. No intergroup differences were noted in the mean corpuscular volume (*p* > 0.05).

**Table 1:** The continuity variables involved in this study are expressed by means (standard error), and the categorical variables are expressed by actual quantities (weighted percentages).

Characteristic	Overall	Quartile 1 [15.63, 56.25]	Quartile 2 (56.25, 66.88]	Quartile 3 (66.88, 76.88]	Quartile 4 (76.88, 100]	*p* value
*N*	7,339	1,882	1,843	1,849	1,765	
Gender, *n* (weighted %)						< 0.0001
Female	3,736 (51.76)	960 (51.80)	873 (46.09)	883 (49.54)	1,020 (58.20)	
Male	3,603 (48.24)	922 (48.20)	970 (53.91)	966 (50.46)	745 (41.80)	
Age, years, *n* (weighted %)						< 0.0001
20–29	1,132 (17.85)	114 (8.48)	208 (11.89)	330 (20.56)	480 (26.71)	
30–39	1,189 (17.19)	208 (13.17)	292 (17.38)	302 (16.32)	387 (20.66)	
40–49	1,296 (20.78)	344 (21.77)	319 (21.52)	321 (20.12)	312 (20.10)	
50–59	1,173 (19.57)	354 (21.61)	312 (20.77)	295 (19.98)	212 (16.81)	
≥60	2,549 (24.61)	862 (34.98)	712 (28.44)	601 (23.01)	374 (15.73)	
Race, *n* (weighted %)						< 0.0001
Non-Hispanic Black	1,258 (9.52)	437 (14.65)	345 (11.26)	292 (8.56)	184 (5.39)	
Mexican American	1,203 (7.41)	279 (6.79)	318 (8.08)	301 (7.13)	305 (7.59)	
Non-Hispanic White	3,903 (74.01)	947 (70.99)	949 (72.83)	1,014 (75.34)	993 (75.85)	
Other race (including multi-racial and other Hispanic)	975 (9.06)	219 (7.57)	231 (7.83)	242 (8.97)	283 (11.17)	
Body mass index, kg/m^2^, *n* (weighted %)						< 0.0001
<25	2,078 (31.01)	202 (10.58)	350 (17.66)	552 (29.65)	974 (57.20)	
25–29.9	2,518 (34.13)	473 (23.85)	654 (34.61)	768 (41.64)	623 (34.18)	
≥30	2,743 (34.86)	1,207 (65.57)	839 (47.73)	529 (28.72)	168 (8.62)	
Education level, *n* (weighted %)						< 0.0001
Above	3,658 (58.85)	671 (41.92)	786 (48.75)	984 (61.30)	1,217 (76.53)	
High school	1,752 (23.84)	502 (30.38)	517 (28.72)	451 (24.76)	282 (14.54)	
Below	1,929 (17.31)	709 (27.70)	540 (22.52)	414 (13.95)	266 (8.94)	
Marital status, *n* (weighted %)						< 0.0001
Married/living with partner	4,532 (65.96)	1,102 (63.09)	1,121 (66.66)	1,177 (66.48)	1,132 (66.95)	
Never married	1,157 (16.33)	191 (10.47)	258 (13.41)	304 (16.45)	404 (22.65)	
Widowed/Divorced/Separated	1,650 (17.72)	589 (26.44)	464 (19.94)	368 (17.07)	229 (10.39)	
Poverty to income ratio, *n* (weighted %)						< 0.0001
<1.3	2,191 (19.12)	716 (27.07)	603 (22.92)	492 (16.92)	380 (12.52)	
1.3–3.49	2,799 (35.21)	742 (39.84)	724 (36.62)	698 (34.30)	635 (31.68)	
≥3.5	2,349 (45.66)	424 (33.10)	516 (40.46)	659 (48.78)	750 (55.80)	
Smoke status, *n* (weighted %)						< 0.0001
Former smoker	1910 (25.66)	546 (26.72)	522 (29.17)	512 (28.14)	330 (19.88)	
Nonsmoker	3,889 (54.15)	601 (31.20)	871 (44.46)	1,038 (55.58)	1,379 (76.70)	
Current smoker	1,540 (20.19)	735 (42.08)	450 (26.37)	299 (16.27)	56 (3.43)	
Alcohol status, *n* (weighted %)						< 0.0001
Former	1,432 (16.42)	552 (26.84)	393 (19.74)	314 (14.02)	173 (8.64)	
Never	954 (10.42)	238 (9.95)	240 (10.18)	219 (9.17)	257 (12.06)	
Mild	2,356 (35.93)	484 (28.10)	545 (33.48)	644 (36.82)	683 (42.57)	
Moderate	1,094 (16.51)	230 (14.18)	258 (14.21)	276 (16.79)	330 (19.71)	
Heavy	1,503 (20.73)	378 (20.93)	407 (22.40)	396 (23.20)	322 (17.02)	
Diabetes, *n* (weighted %)						< 0.0001
No	6,167 (88.32)	1,230 (70.81)	1,542 (86.71)	1,683 (93.69)	1,712 (97.06)	
Yes	1,172 (11.68)	652 (29.19)	301 (13.29)	166 (6.31)	53 (2.94)	
Hypertension, *n* (weighted %)						< 0.0001
No	4,235 (63.74)	612 (37.12)	933 (54.34)	1,209 (68.32)	1,481 (85.78)	
Yes	3,104 (36.26)	1,270 (62.88)	910 (45.66)	640 (31.68)	284 (14.22)	
Hyperlipidemia, *n* (weighted %)						< 0.0001
No	1,840 (26.34)	201 (9.75)	303 (15.19)	524 (27.56)	812 (45.73)	
Yes	5,499 (73.66)	1,681 (90.25)	1,540 (84.81)	1,325 (72.44)	953 (54.27)	
Phenotypic age, years	42.67 (0.45)	52.37 (0.51)	46.03 (0.54)	40.93 (0.62)	34.74 (0.68)	< 0.0001
Phenotypic age acceleration	−4.72 (0.13)	−0.34 (0.22)	−3.95 (0.21)	−5.57 (0.19)	−7.63 (0.16)	< 0.0001
Refrige glucose, mmol/L	5.39 (0.03)	6.17 (0.07)	5.42 (0.05)	5.20 (0.03)	4.98 (0.02)	< 0.0001
Alkaline phosphatase, U/L	66.77 (0.35)	74.37 (0.73)	69.55 (0.62)	65.49 (0.48)	60.36 (0.58)	< 0.0001
Albumin, g/L	42.77 (0.08)	41.81 (0.12)	42.58 (0.13)	42.94 (0.08)	43.45 (0.12)	< 0.0001
Creatinine, μmol/L	78.20 (0.50)	81.65 (1.01)	80.69 (0.83)	77.27 (0.56)	74.66 (0.64)	< 0.0001
C-reactive protein, mg/dL	0.37 (0.01)	0.59 (0.03)	0.40 (0.02)	0.32 (0.02)	0.22 (0.01)	< 0.0001
WBC, 1,000 cells/μL	7.14 (0.04)	8.10 (0.08)	7.37 (0.06)	6.92 (0.05)	6.50 (0.04)	< 0.0001
Lymphocyte percent, %	30.23 (0.16)	29.29 (0.21)	30.01 (0.27)	30.29 (0.23)	31.02 (0.23)	< 0.0001
Mean cell volume, fL	89.27 (0.23)	89.08 (0.27)	89.27 (0.30)	89.03 (0.27)	89.64 (0.24)	0.05
Red cell distribution width, %	12.75 (0.03)	13.08 (0.04)	12.80 (0.04)	12.73 (0.04)	12.49 (0.03)	< 0.0001

The One-way ANOVA applies to continuity variables and Chi-square test applies to categorical variables; Q1, the first quartile; Q2, the second quartile; Q3, the third quartile; Q4, the fourth quartile; ref, reference; Life’s Essential 8 quartile ranges: Quartile 1 = 15.63–56.25; Quartile 2 = 56.26–66.88; Quartile 3 = 66.89–76.88; and Quartile 4: 76.88–100.

### Association analysis between Life’s Essential 8 and Phenotypic Age Acceleration in American adults

The generalized linear regression weighted model for LE8 and PhenoAgeAccel demonstrated (Table 2) that in the crude model, compared to the lowest quartile of LE8, there was a more significant negative correlation between LE8 Q2 (*p* < 0.0001), Q3 (*p* < 0.0001), Q4 (*p* < 0.0001), and PhenoAgeAccel, with the trend test showing significance (*p* < 0.05). In the fully adjusted model (Model 2), there was a more pronounced negative correlation between LE8 Q2 (*p* = 0.003), Q3 (*p* = 0.003), Q4 (*p* = 0.001), and PhenoAgeAccel, compared to the lowest quartile of LE8, with the trend test being significant (*p* < 0.001).

**Table 2:** Crude model is the unadjusted model.

	Crude model		Model 1		Model 2	
	95% CI	*p*	95% CI	*p*	95% CI	*p*
Life’s Essential 8	−0.2 (−0.21, −0.18)	<0.0001	−0.14 (−0.17,−0.11)	<0.0001	−0.1 (−0.13, −0.07)	<0.001
Q1	ref		ref		ref	
Q2	−3.61 (−4.15, −3.07)	<0.0001	−2.74 (−3.41, −2.08)	<0.0001	−1.77 (−2.56, −0.98)	0.003
Q3	−5.22 (−5.83, −4.62)	<0.0001	−3.43 (−4.27, −2.58)	<0.0001	−2.08 (−2.96, −1.20)	0.003
Q4	−7.29 (−7.80, −6.78)	<0.0001	−4.34 (−5.24, −3.43)	<0.0001	−2.82 (−3.82, −1.83)	0.001
*p* for trend		<0.0001		<0.0001		<0.001

Model 1 adjusted for age, gender, race, poverty to income ratio, education level, BMI, marital status, smoke status, and alcohol status. Model 2 adjusted for age, gender, race, poverty to income ratio, education level, BMI, marital status, smoke status, alcohol status, diabetes, hypertension, and hyperlipidemia.

Results from the gender subgroup generalized linear regression weighted model indicated (Table 3) that in the fully adjusted model for males (Model 2), compared to the lowest quartile of LE8, there was a more pronounced negative correlation between LE8 Q2 (*p* = 0.02), Q3 (*p* = 0.01), Q4 (*p* = 0.002), and PhenoAgeAccel, with the trend test indicating significance (*p* < 0.001). In the fully adjusted model for females (Model 2), compared to the lowest quartile of LE8, LE8 Q2 (*p* = 0.003), Q3 (*p* = 0.01), and Q4 (*p* = 0.002) were significantly negatively correlated with PhenoAgeAccel, with the trend test showing significance (*p* = 0.001).

**Table 3:** Crude model is the unadjusted model.

Gender: Male	Crude model		Model 1		Model 2	
	95% CI	*p*	95% CI	*p*	95% CI	*p*
Life’s Essential 8	−0.18 (−0.20, −0.16)	<0.0001	−0.14 (−0.17, −0.11)	<0.0001	−0.09 (−0.12, −0.06)	<0.001
Q1	ref		ref		ref	
Q2	−3 (−3.82, −2.18)	<0.0001	−2.16 (−2.97, −1.35)	<0.001	−1.15 (−2.07, −0.23)	0.02
Q3	−4.52 (−5.34, −3.69)	<0.0001	−3.11 (−3.97, −2.25)	<0.0001	−1.7 (−2.62, −0.78)	0.01
Q4	−6.4 (−7.26, −5.53)	<0.0001	−4.14 (−5.19, −3.09)	<0.0001	−2.41 (−3.46, −1.35)	0.002
*p* for trend		<0.0001		<0.0001		<0.001
Gender: Female	Crude model		Model 1		Model 2	
	95% CI	*p*	95%CI	*p*	95%CI	*p*
Life’s Essential 8	−0.21 (−0.22, −0.19)	<0.0001	−0.15 (−0.20, −0.11)	<0.0001	−0.12 (−0.16, −0.07)	<0.001
Q1	ref		ref		ref	
Q2	−4.29 (−4.99, −3.59)	<0.0001	−3.41 (−4.36, −2.47)	<0.0001	−2.45 (−3.58, −1.33)	0.003
Q3	−5.93 (−6.77, −5.09)	<0.0001	−4.06 (−5.43, −2.70)	<0.001	−2.74 (−4.24, −1.25)	0.01
Q4	−7.96 (−8.64, −7.28)	<0.0001	−4.97 (−6.32, −3.63)	<0.0001	−3.57 (−5.06, −2.08)	0.002
*p* for trend		<0.0001		<0.0001		0.001

Model 1 adjusted for age, race, poverty to income ratio, education level, BMI, marital status, smoke status, and alcohol status. Model 2 adjusted for age, race, poverty to income ratio, education level, BMI, marital status, smoke status, alcohol status, diabetes, hypertension, and hyperlipidemia. Q1, The first quartile; Q2, The second quartile; Q3, The third quartile; Q4, The fourth quartile; and ref, reference.

### Restricted cubic spline visualization

**Figure 2:**
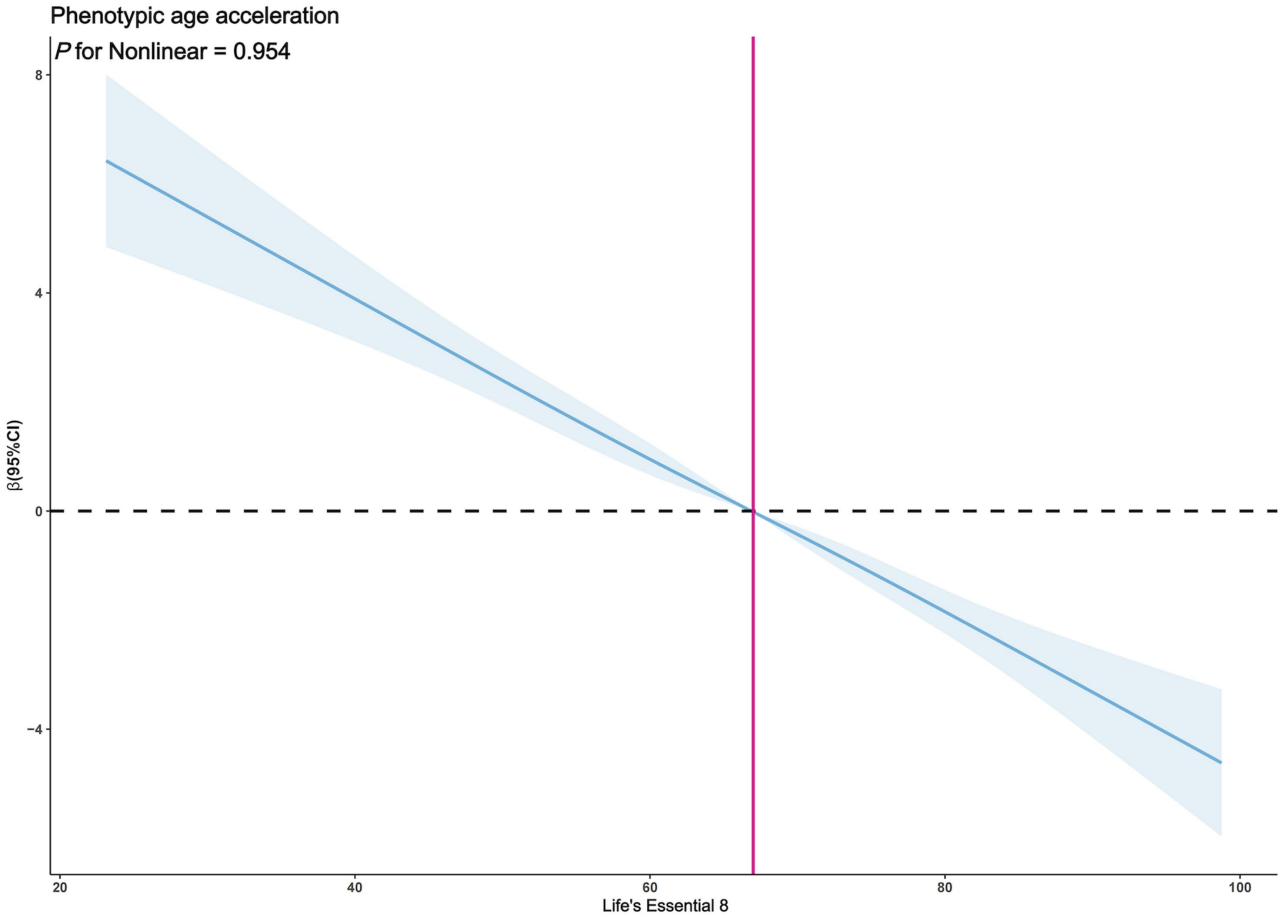
Restricted cubic spline plot model. The adjusted restricted cubic spline plot model shows an association between PhenoAgeAccel and LE8 among all participants. The model was adjusted for age, gender, race, poverty to income ratio, education level, BMI, marital status, smoke status, alcohol status, diabetes, hypertension, and hyperlipidemia. The blue solid line and the blue shaded area represent the estimated regression coefficient Beta and its 95% confidence interval.

**Figure 3:**
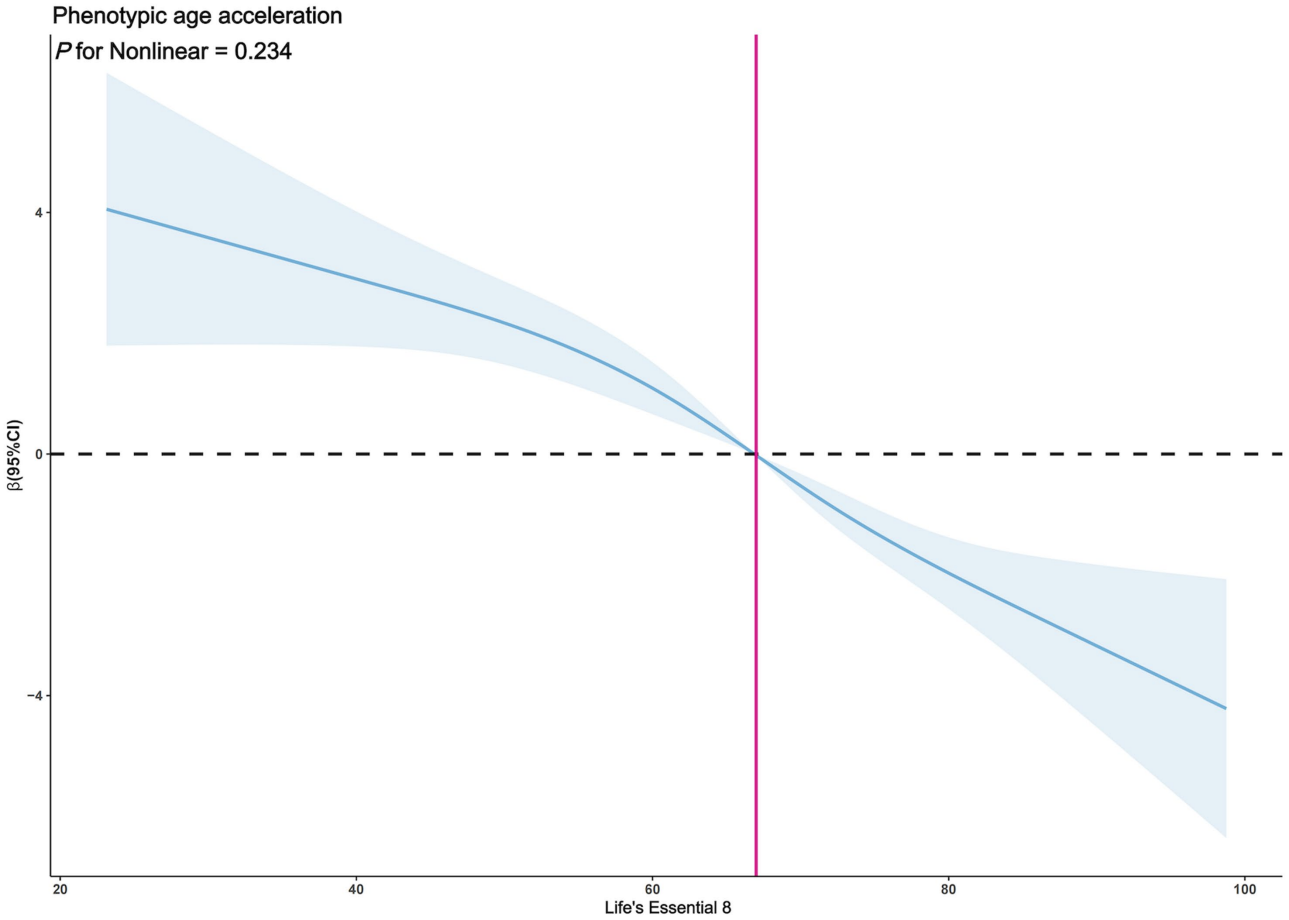
Restricted cubic spline plot model (Male). The adjusted restricted cubic spline plot model shows an association between PhenoAgeAccel and LE8 among all participants. The model was adjusted for age, race, poverty to income ratio, education level, BMI, marital status, smoke status, alcohol status, diabetes, hypertension, and hyperlipidemia. The blue solid line and the blue shaded area represent the estimated regression coefficient Beta and its 95% confidence interval.

**Figure 4:**
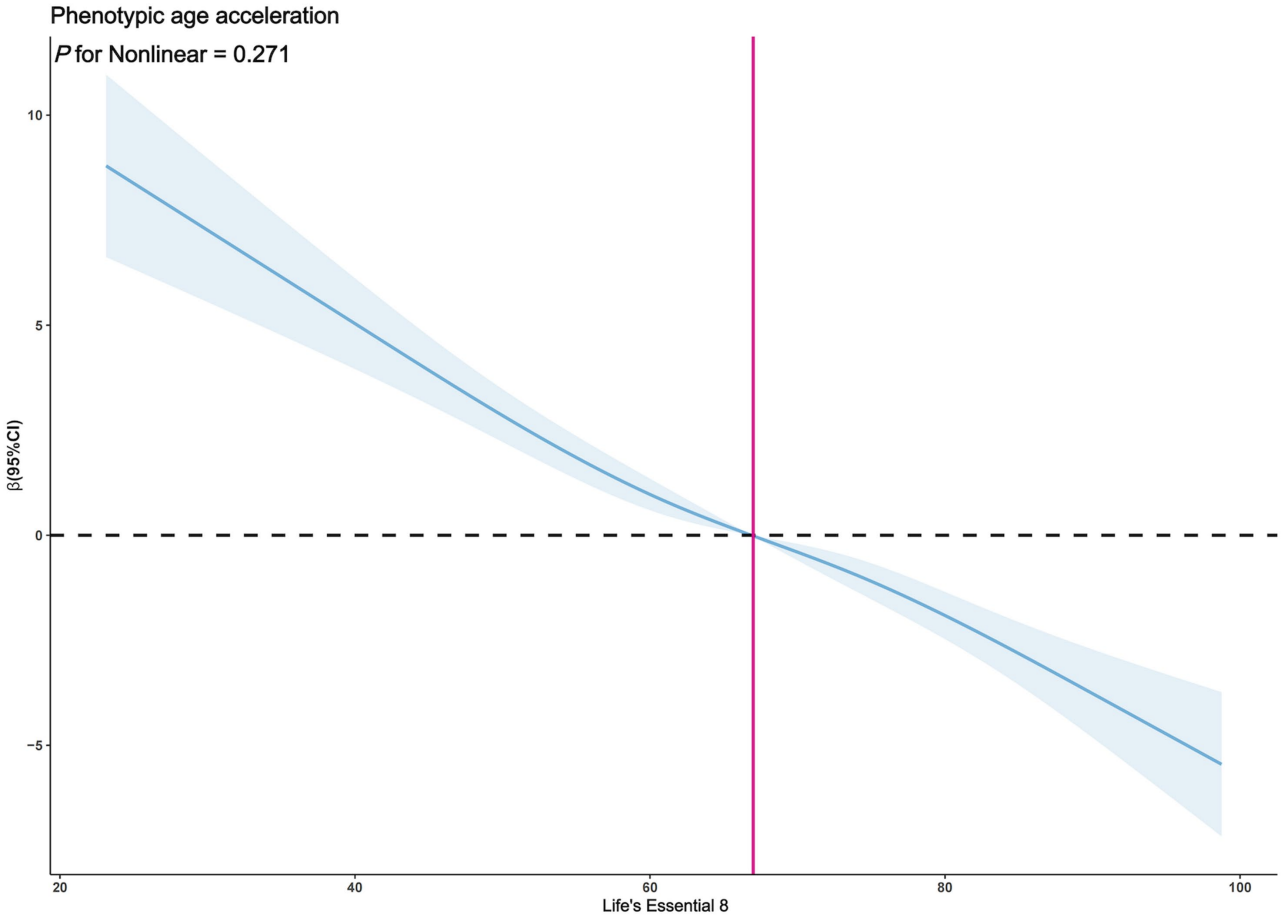
Restricted cubic spline plot model (Female). The adjusted restricted cubic spline plot model shows an association between PhenoAgeAccel and LE8 among all participants. The model was adjusted for age, race, poverty to income ratio, education level, BMI, marital status, smoke status, alcohol status, diabetes, hypertension, and hyperlipidemia. The blue solid line and the blue shaded area represent the estimated regression coefficient Beta and its 95% confidence interval.

The exploration of whether there exists a non-linear correlation between LE8 and PhenoAgeAccel was further conducted through restricted cubic spline (RCS) graphs. The results presented in Figure 2 demonstrate no significant non-linear trends between LE8 and PhenoAgeAccel (*p* for Nonlinear = 0.954). The sub-group analysis based on gender reveals that upon controlling the variables through a fully adjusted linear regression model (Model 2) for males, there were no notable non-linear trends between male LE8 and PhenoAgeAccel as depicted in Figure 3 (*p* for Nonlinear = 0.234). Similarly, Figure 4 illustrates an insignificant non-linear trend between female LE8 and PhenoAgeAccel (*p* for Nonlinear = 0.271).

### Interaction effect test

The results of the interaction effect test shown in Table 4 indicate that the stratification variables chosen in this study, which include age, gender, ethnicity, education level, marital status, poverty-income ratio, smoking status, drinking status, hypertension, and hyperlipidemia, did not produce an interaction effect on the relationship between LE8 and PhenoAgeAccel (*p* for interaction > 0.05). However, body mass index (BMI) and diabetes did exhibit interaction effects on the correlation between LE8 and PhenoAgeAccel (*p* for interaction < 0.05), suggesting that the influence of LE8 on PhenoAgeAccel might vary depending on an individual’s BMI and whether they have diabetes.

**Table 4:** Adjusted for age, gender, race, poverty to income ratio, education level, BMI, marital status, smoke status, alcohol status, diabetes, hypertension, and hyperlipidemia.

	Q1	Q2	*p*	Q3	*p*	Q4	*p*	*p* for trend	*p* for interaction
Gender									0.15
Female	ref	−2.45 (−3.58, −1.33)	0.003	−2.74 (−4.24, −1.25)	0.01	−3.57 (−5.06, −2.08)	0.002	0.001	
Male	ref	−1.15 (−2.07, −0.23)	0.02	−1.7 (−2.62, −0.78)	0.01	−2.41 (−3.46, −1.35)	0.002	<0.001	
Age									0.61
20–29	ref	−0.99 (−2.82, 0.83)	0.24	−1.65 (−2.67, −0.62)	0.01	−2.36 (−3.75, −0.97)	0.004	0.01	
30–39	ref	−0.93 (−1.90, 0.04)	0.06	−1.42 (−2.77, −0.07)	0.04	−2 (−3.53, −0.47)	0.02	0.02	
40–49	ref	−2.59 (−4.22, −0.95)	0.01	−2.44 (−4.12, −0.77)	0.01	−4.04 (−5.74, −2.34)	<0.001	<0.001	
50–59	ref	−1.82 (−3.08, −0.56)	0.01	−2.05 (−3.67, −0.44)	0.02	−2.66 (−4.43, −0.89)	0.01	0.01	
≥60	ref	−1.69 (−2.77, −0.61)	0.01	−2.45 (−3.55, −1.36)	<0.001	−2.73 (−4.30, −1.17)	0.004	0.002	
Race									0.12
Non-Hispanic Black	ref	−2.76 (−5.48, −0.04)	0.05	−2.79 (−5.76, 0.19)	0.06	−4.28 (−7.46, −1.09)	0.02	0.01	
Mexican American	ref	−1.11 (−2.46, 0.25)	0.09	−1.09 (−2.72, 0.54)	0.15	−2.49 (−4.07, −0.91)	0.01	0.01	
Non-Hispanic White	ref	−1.76 (−2.56, −0.96)	0.002	−2.04 (−2.93, −1.15)	0.001	−2.62 (−3.78, −1.46)	0.001	0.001	
Other race	ref	−0.27 (−2.23, 1.69)	0.75	−1.72 (−3.45, 0.01)	0.05	−3 (−5.41, −0.59)	0.02	0.02	
Body mass index									0.03
<25	ref	−0.85 (−3.75, 2.05)	0.5	−2.35 (−4.81, 0.11)	0.06	−2.97 (−5.49, −0.44)	0.03	0.01	
25–29.9	ref	−2.84 (−3.97, −1.70)	<0.001	−2.44 (−3.75, −1.12)	0.004	−3.03 (−4.69, −1.37)	0.004	0.01	
≥30	ref	−1.5 (−2.44, −0.55)	0.01	−1.99 (−2.92, −1.07)	0.002	−2.65 (−3.77, −1.53)	0.001	<0.001	
Education level									0.24
Above	ref	−1.96 (−2.95, −0.97)	0.003	−2.08 (−3.03, −1.13)	0.002	−2.61 (−3.69, −1.53)	0.001	<0.001	
High school	ref	−2.37 (−3.16, −1.58)	<0.001	−2.34 (−3.54, −1.14)	0.003	−3.77 (−5.23, −2.31)	<0.001	<0.001	
Below	ref	−0.94 (−2.79, 0.91)	0.26	−1.98 (−4.27, 0.31)	0.08	−2.97 (−5.35, −0.58)	0.02	0.02	
Marital status									0.22
Married/living with partner	ref	−2.31 (−3.34, −1.27)	0.002	−2.12 (−3.28, −0.96)	0.004	−2.91 (−4.14, −1.69)	0.001	0.001	
Never married	ref	−0.75 (−2.56, 1.05)	0.35	−2.44 (−3.87, −1.00)	0.01	−3 (−4.78, −1.22)	0.01	0.01	
Widowed/Divorced/Separated	ref	−0.88 (−2.52, 0.77)	0.24	−1.91 (−4.01, 0.19)	0.07	−2.9 (−5.24, −0.56)	0.02	0.02	
Poverty to income ratio									0.19
<1.3	ref	−1.58 (−3.15, −0.02)	0.05	−2.13 (−3.45, −0.80)	0.01	−3.57 (−5.46, −1.68)	0.004	0.002	
1.3–3.49	ref	−1.64 (−2.69, −0.59)	0.01	−1.98 (−3.21, −0.75)	0.01	−2.43 (−3.98, −0.88)	0.01	0.01	
≥3.5	ref	−1.89 (−3.02, −0.75)	0.01	−2.14 (−3.16, −1.13)	0.002	−2.98 (−4.29, −1.68)	0.001	<0.001	
Smoke status									0.16
Former smoker	ref	−2.56 (−3.86, −1.25)	0.003	−2.42 (−4.11, −0.72)	0.01	−3.75 (−5.51, −1.98)	0.002	0.003	
Nonsmoker	ref	−2 (−2.93, −1.07)	0.002	−2.49 (−3.16, −1.81)	<0.001	−2.93 (−3.87, −2.00)	<0.001	<0.001	
Current smoker	ref	−0.8 (−2.20, 0.60)	0.21	−1.18 (−2.70, 0.34)	0.11	−2.52 (−4.19, −0.86)	0.01	0.03	
Alcohol status									0.34
Former	ref	−2.33 (−4.26, −0.40)	0.02	−2.59 (−4.58, −0.61)	0.02	−4.04 (−6.51, −1.58)	0.01	0.01	
Never	ref	−1.85 (−3.68, −0.02)	0.05	−2.7 (−4.92, −0.48)	0.02	−3.58 (−5.62, −1.54)	0.004	0.003	
Mild	ref	−1.96 (−3.26, −0.66)	0.01	−2.12 (−3.43, −0.81)	0.01	−2.7 (−4.18, −1.22)	0.003	0.004	
Moderate	ref	−1.29 (−2.87, 0.29)	0.1	−1.87 (−3.51, −0.22)	0.03	−2.63 (−4.43, −0.83)	0.01	0.01	
Heavy	ref	−0.92 (−1.76, −0.08)	0.04	−1.34 (−2.18, −0.49)	0.01	−1.96 (−3.06, −0.86)	0.003	0.001	
Diabetes									< 0.001
No	ref	−1.12 (−1.78, −0.46)	0.01	−1.31 (−2.10, −0.52)	0.01	−2.11 (−3.00, −1.22)	0.002	0.002	
Yes	ref	−3.63 (−6.26, −1)	0.02	−6.24 (−9.22, −3.26)	0.003	−6.23 (−10.23, −2.24)	0.01	<0.001	
Hypertension									0.16
No	ref	−2.07 (−2.94, −1.19)	0.002	−3 (−3.69, −2.30)	<0.001	−3.81 (−5.44, −2.19)	0.002	<0.0001	
Yes	ref	−1.33 (−2.41, −0.26)	0.02	−1.37 (−2.60, −0.14)	0.04	−2.1 (−3.30, −0.89)	0.01	0.005	
Hyperlipidemia									0.06
No	ref	−1.88 (−2.55, −1.21)	<0.001	−2.12 (−3.04, −1.21)	0.002	−2.64 (−3.77, −1.51)	0.002	0.001	
Yes	ref	−0.74 (−2.54, 1.05)	0.34	−1.57 (−3, −0.13)	0.04	−2.86 (−4.40, −1.33)	0.005	0.001	

Q1, The first quartile; Q2, The second quartile; Q3, The third quartile; and Q4, The fourth quartile; ref, reference.

## Discussion

This research based on NHANES data from 2007 to 2010 delves into the relationship between LE8 and PhenoAgeAccel. It found a significant negative correlation between LE8 and PhenoAgeAccel when considering a multitude of population variables, a point corroborated across different gender subgroups. However, the non-linear trend in this relationship was not pronounced, suggesting a stable linear trend that implies a direct and continuous relationship between LE8 and PhenoAgeAccel, unaffected by certain specific thresholds. Furthermore, aside from body mass index and diabetes, no other variables exhibited significant interactive effects with LE8 and PhenoAgeAccel. This might hint that the impact of LE8 on PhenoAgeAccel varies with individual body mass index and diabetic conditions. In conclusion, this negative correlation potentially indicates that higher levels of LE8 are associated with a slower PhenoAgeAccel process, but this necessitates further research to pinpoint the underlying biological mechanisms and clinical implications.

Life’s Essential 8, the latest definition of ideal cardiovascular health set by the American Heart Association, encompasses four health behaviors (diet, physical activity, nicotine exposure, and sleep) and four health factors (body mass index, blood pressure, blood sugar, and cholesterol levels) (9). Studies affirm its tight correlation with reduced risks of adverse health outcomes. Higher LE8 scores indicate better cardiovascular health and lower coronary heart disease risks across different populations (26). In the Women’s Health Initiative cohort of postmenopausal women, the average LE8 score was moderate, with about a quarter having good cardiovascular health (27). LE8 scores can be accurately estimated using routinely collected parameters like demographics, body mass index, smoking status, blood pressure, and medical history, even when some metrics are missing (28). A recent study unveiled a significant negative correlation between LE8 scores and cardiovascular disease mortality and all-cause mortality risk in middle-aged and older Finnish men. The research established that an increase of 50 points in the LE8 score lowers the risks by 17 and 14% for cardiovascular disease mortality and all-cause mortality respectively, compared to the lowest quartile (29). Another recent study noted a 23% increased risk of hypertension over 6 years associated with lower LE8 scores (30). Research demonstrated that the interaction of LE8 with genetic susceptibility significantly affects health outcomes, with high LE8 levels considerably reducing adverse cardiovascular disease event risks in individuals with high genetic predispositions; notably, ideal cardiovascular health can decrease the risk by 74%. Moreover, nearly 70% of health outcomes and 51.2% of the associations between genetic susceptibility and health outcomes can be attributed to LE8 (24). In sum, these studies provide compelling evidence that achieving and maintaining the ideal cardiovascular health defined by LE8 can offer life-changing health benefits, especially for individuals with higher genetic risks.

Epigenetic age acceleration, the differential between an individual’s biological and actual age, has emerged as a central focus in aging research. Multiple studies have unveiled its significant associations with adverse health outcomes and heightened risks of early mortality (31–33), influenced by a complex array of factors including socio-psychological stress and early life adversities, which have been proven to be linked with advanced epigenetic age (14, 34). This process begins early in life, profoundly impacted by growth experiences. For instance, lower birth weights and poor maternal nutrition during pregnancy have been associated with higher epigenetic age in young adults, particularly in males (35). Novel research methodologies are striving to enhance our understanding of what epigenetic age acceleration represents at the biological level, including the establishment of probabilistic models to elucidate how cellular level methylation variations lead to accelerated or deviated estimates of epigenetic age (36). Moreover, PhenoAgeAccel, a novel biomarker for aging, has been linked with lifestyle factors such as body mass index, fat content, and physical activity levels (37, 38). Recent findings show that regular physical activity can decelerate epigenetic aging by slowing immune aging and reducing cardiovascular risks (39). This supports the current research, where LE8, principally constituted of health behaviors and factors including physical activity, body mass index, and cholesterol levels, plays a vital role in influencing the human aging process. PhenoAgeAccel not only facilitates a deeper understanding of aging biology and the development of aging-related morbidity but also uncovers how lifestyle modifications can influence the speed of biological aging when combined with LE8. It is evident that epigenetic age acceleration is a multifaceted and complex phenomenon, associated with adverse health outcomes and influenced by early life experiences, offering a window to comprehend and explore the utility of epigenetic clocks in predicting health and longevity, thus presenting a valuable direction for future research and interventions.

It is widely recognized that physical activity positively influences the resistance to the processes of aging. Larasati (40) underscored the myriad benefits of physical exercise for older individuals, encompassing improvements in cardiovascular health, reduction in the risk of chronic diseases, and upliftment of mental health levels. Glynn (41) further indicated that sustained physical training could markedly decelerate the physiological impacts of aging, thereby fostering successful aging. Research conducted by Rogers (42) discovered that high-intensity physical workouts, especially strenuous exercises, could slow down the weakening process in older individuals. Pabianek (43) also affirmed the importance of physical training, emphasizing its role in reducing age-related muscle loss and lowering the risk of chronic diseases. These studies collectively corroborate the pivotal role of physical exercise in delaying the aging process and promoting healthy aging.

In LE8, dietary health follows HEI-2015, establishing a correlation between HEI-2015 and aging-associated outcomes. Abigail et al. (44) emphasized a dose-dependent relationship between diet quality and both longevity and successful aging. Ma (45) revealed a positive correlation between HEI-2015 and the plasma levels of the anti-aging protein S-Klotho, suggesting a potential link between healthy eating and anti-aging attributes. Research by Fan et al. (46) established a negative correlation between higher HEI-2015 scores and a reduced likelihood of physical frailty in American older individuals. These findings collectively suggest that adhering to healthy dietary patterns could exert beneficial impacts on aging-related outcomes.

In LE8, nicotine exposure is primarily assessed through smoking habits, associating it with accelerated aging. Park et al. (47) found a significant relationship between genetically predicted and observed smoking behaviors and aging phenotypes. Linli et al. (48) demonstrated that smoking correlates with accelerated brain aging, influenced by smoking characteristics like duration and intensity. Research by Laksmi et al. (49) stated that smoking and low levels of superoxide dismutase (SOD) are risk factors for premature aging in women. These studies signify the association of nicotine exposure with biological aging, highlighting the necessity to augment awareness regarding this connection. Previous research unequivocally illustrates an interdependent relationship between sleep quality and aging. Casagrande’s (50) study unveiled the linkage between sleep disturbances and cognitive impairments, particularly accentuated in Alzheimer’s patients. Research by Gkotzamanis et al. (51) discovered a negative correlation between poor sleep quality and elongated sleep duration with healthy aging trajectories. Moreover, Tibon et al. (52) verified the connection between brain network dynamic alterations with sleep quality and cognitive performances, reinforcing their relation to aging. These investigations jointly underline the imperative of maintaining good sleep quality in the healthy aging trajectory, pinpointing the close ties between sleep quality, cognitive functions, and brain network dynamics. A connection exists between Body Mass Index (BMI) and aging. Lundgren et al. (53) found that higher BMIs correlate positively with accelerated epigenetic aging. Santos et al. (54) discussed the molecular mechanisms connecting obesity with aging, emphasizing the similarities in metabolic dysfunction and the potential for obesity to speed up aging. Tam et al. (55) further affirmed the similarities in phenotypes and comorbidities between obesity and aging, including compromised genome integrity, mitochondrial dysfunction, and systemic inflammation. Reyes-Farias (56) focused on the functional impairments in white adipose tissue in obesity and aging, highlighting the role of chronic low-grade inflammation in mediating aging. These studies demonstrate the interconnected nature of body mass and aging, bearing similar causal features, and physiological mechanisms. Blood pressure exhibits alterations with advancing age, generally characterized by an increase in systolic pressure while diastolic pressure remains stable or even reduces, potentially associating these periodic dynamic changes in blood pressure with biological aging. Research by Xiao et al. (57) disclosed that higher systolic and pulse pressures are related to accelerated epigenetic aging. Dintica et al. (58) proposed that elevated blood pressure during middle age associates with premature brain aging. Omboni et al. (59) documented alterations in blood pressure patterns with aging, including increased variability and prevalence of hypertension. Farron et al. (60) stressed that higher systolic and lower diastolic pressures independently correlate with poorer cognitive function in older individuals. These studies spotlight the crucial role of blood pressure management in the aging process and biological aging, and its impact on various aspects of health. Blood glucose levels have consistently been a focal point in aging research. Bahour et al. (61) found that individuals with type 2 diabetes have a higher physiological age compared to those without the disease. Selvin et al. (62) observed considerable variations in blood glucose patterns among older adults without diabetes, including lower readings. Research by Sorlí et al. (63) noticed age-dependent relationships between genetic variations associated with fasting glucose and actual age. Edqvist et al. (64) reported higher levels of HbA1c throughout adulthood in individuals diagnosed with type 1 diabetes at a younger age. These studies hint that blood glucose levels may be related to phenotypic age, suggesting a potential connection between blood glucose control and the aging process (65). Blood lipid levels dynamically change as age advances, playing a critical role in regulating aging and lifespan through lipid metabolism and signaling pathways. Lefèvre-Arbogast et al. (66) associated specific lipids involved in membrane fluidity and myelin formation with subsequent cognitive decline in older individuals. Research by Cesare et al. (67) detailed the variations in blood metabolites and lipid concentrations, correlatives, and ratios with age, indicating the remodeling of lipid metabolism with aging. Slade et al. (68) identified age and gender correlations with various lipid species, subclasses, and categories, emphasizing the potential utilization of lipid phenotypes as biomarkers reflecting age-related changes in lipids and cardiovascular risks. Mutlu et al. (65) delineated the pivotal role of lipids in governing aging and lifespan, underlining the connections between lipid metabolism, signaling, and lifespan regulation. These studies collaboratively emphasize the vital role of lipids in aging and lifespan control, alongside their potential as biomarkers, highlighting the significance of lipid metabolism and signaling pathways in aging and lifespan regulation. In conclusion, the aforementioned research grounds on the nine constituent factors in LE8, inclusive of four health behaviors (diet, physical exercise, nicotine exposure, and sleep) and four health factors (BMI, blood pressure, blood glucose, and blood lipids) linked to aging, furnishes further theoretical backing and data support for this study. It accentuates the central roles of various lifestyle and health factors in the aging process.

This study leveraged the NHANES data from 2007 to 2010 to explore the potential relationship between LE8 and PhenoAgeAccel in depth. The findings underscored the central role of healthy lifestyles and favorable physiological indicators in slowing down the aging process. This not only provides a scientific basis for formulating more targeted health policies and intervention measures but also paves new pathways for public health education, facilitating a deeper understanding and awareness of LE8 among the public, guiding them toward healthier lifestyles. However, the study had several limitations: (1) The study utilized NHANES data, characterized by a cross-sectional study design, which cannot establish causality between LE8 and PhenoAgeAccel, only reflecting their correlation. Therefore, the results cannot exclude the influence of other potential confounding or mediating factors, making it unable to infer the long-term impact of LE8 on PhenoAgeAccel accurately. To verify the causal mechanism between the two, it is necessary to carry out more randomized controlled trials or longitudinal cohort studies. (2) Cross-sectional studies often rely on self-reported data, possibly introducing memory and reporting biases. In this study, some components of LE8 (such as diet and physical exercise) might be affected by these biases. (3) As the study relied on existing data from the database, we could not control all variables potentially affecting the outcomes, meaning that there might be the presence of confounding variables, possibly influencing our understanding of the relationship between LE8 and PhenoAgeAccel. (4) The study used NHANES data, a survey based on a representative sample of non-institutionalized residents in the United States. However, the United States population is diverse and heterogeneous, and factors like different races, regions, cultures, and lifestyles might affect the relationship between LE8 and PhenoAgeAccel. Hence, the results cannot be directly generalized to the populations of other countries or regions, necessitating more cross-national or cross-regional comparative studies.

Despite these limitations, this study still lays an essential foundation, offering directions for future research. It emphasizes the significance of healthy lifestyles and good physiological indicators in slowing down the aging process, offering valuable insights for public health policies and education. Through further research, we can better understand the relationship between LE8 and PhenoAgeAccel and how to leverage this understanding to formulate more effective health policies and intervention measures.

## Conclusion

This study, based on NHANES data from 2007 to 2010, has revealed a significant negative correlation between LE8 and PhenoAgeAccel, emphasizing the importance of maintaining a healthy lifestyle in slowing down the biological aging process. Despite the limitations posed by the study’s design and geographical constraints, these findings provide a scientific basis for the development of public health policies focused on healthy lifestyle practices. Future research should further investigate the causal mechanisms underlying the relationship between LE8 and PhenoAgeAccel and consider cross-cultural comparisons to enhance our understanding of healthy aging.

## Data availability statement

The datasets presented in this study can be found in online repositories. The names of the repository/repositories and accession number(s) can be found at: https://wwwn.cdc.gov/nchs/nhanes/continuousnhanes/default.aspx?BeginYear=2009.

## Ethics statement

The studies involving humans were approved by National Center for Health Statistics Home. The studies were conducted in accordance with the local legislation and institutional requirements. The participants provided their written informed consent to participate in this study.

## Author contributions

DW: Writing – original draft, Writing – review & editing. CQ: Data curation, Writing – review & editing. PH: Data curation, Formal analysis, Investigation, Writing – review & editing. XG: Data curation, Writing – review & editing. JiaZ: Writing – review & editing. YS: Investigation, Methodology, Writing – review & editing. ZR: Data curation, Investigation, Writing – review & editing. JieZ: Supervision, Writing – review & editing, Writing – original draft.
